# Osteoporosis medication adherence – are the myths true or do patients take their Calcichew®?

**DOI:** 10.1186/1753-6561-6-S4-P36

**Published:** 2012-07-09

**Authors:** G Burke, G McCarthy

**Affiliations:** 1 School of Medicine, Royal College of Surgeons in Ireland, Ireland; 2 Molecular & Cellular Therapeutics, Royal College of Surgeons in Ireland, Ireland

## Introduction & objectives

Osteoporosis is defined as a progressive systemic disease characterised by low bone mass and architectural deterioration of bone tissue, with consequent increased bone fragility. The disease is predominately a disease of postmenopausal women and those prescribed long-term oral corticosteroids, but may occur in all populations and ages. A large number of patients are treated for osteoporosis in Ireland and adherence is a major issue in it's management. Poor adherence to osteoporosis treatment has been well documented and is a widely recognised problem, resulting in a huge burden on patients and the healthcare system. In particular, poor adherence results in an increased number of fractures and consequent pain and disability. The authors noted that patients frequently stated they did not wish to have their osteoporosis medication dispensed. In practice, a number of factors affect adherence to medication and there are numerous ways it can be improved. The objectives of this study were to ascertain the extent of failures to refill prescriptions for osteoporosis medications in the community setting, and to make recommendations.

## Methods

An extensive literature review was carried out to obtain an overview of the topic under research. This was also used for comparative purposes. A retrospective review of prescription records of 200 patients (N=200 F=168 M=32) who were prescribed osteoporosis medications from January 2011- June 2011 was completed. Data from 17 pharmacies with mixed demographics in the Munster and south Leinster regions was used. The details recorded were the dates of refill, the number of refills per six months, number of concurrent medications including other osteoporosis treatments, gender, age and whether the patient paid for the medication or obtained it free of charge. Adherence was classified using the medication possession ratio (MPR).

## Results & conclusions

There are a large and increasing number of osteoporosis medications on the market in Ireland. As described in the literature, adherence was sub-optimal. The adherence to oral calcium and vitamin D supplements was 48%, bisphosphonates 69%, Selective Oestrogen Receptor Modulator (SERM) 84%, Dual Action Bone Agent (DABA) Strontium 81%. There were significant variations in adherence between the different classes of drugs and variations within the same class. This varied with patient demographics.

